# Editorial: Inflammation in Healing and Regeneration of Cutaneous Wounds

**DOI:** 10.3389/fimmu.2021.806687

**Published:** 2021-11-19

**Authors:** Allison J. Cowin, Ardeshir Bayat, Rachael Z. Murray, Zlatko Kopecki

**Affiliations:** ^1^ Future Industries Institute and Science Technology Engineering and Maths (STEM) Academic Unit, University of South Australia, Adelaide, SA, Australia; ^2^ Clinical and Health Sciences, University of South Australia, Adelaide, SA, Australia; ^3^ Plastic and Reconstructive Surgery Research, Centre for Dermatology Research, National Institute for Health Research (NIHR) Manchester Biomedical Research Centre, University of Manchester, Manchester, United Kingdom; ^4^ South African Medical Research Council (MRC-SA) Wound Healing Unit, Division of Dermatology, University of Cape Town, Cape Town, South Africa; ^5^ School of Biomedical Sciences, Faculty of Health, Queensland University of Technology, Brisbane, QLD, Australia

**Keywords:** inflammation, wounds, cutaneous healing, tissue regeneration, scars

Healing and regeneration of wounds is a complex process that has at its core a functional inflammatory response which initiates the repair process and fights against infective pathogens. Inflammation is an evolutionarily developed process that facilitates host responses to tissue injury, and is critical in reestablishment of skin homeostasis, wound healing, and tissue regeneration. The tightly regulated inflammatory process driven by innate immune responses and numerous cell types including neutrophils, macrophages, mast cells, and various leukocytes facilitates healthy wound healing and tissue regeneration with minimal scarring consequences. However, many chronic conditions can contribute to a dysregulated and prolonged inflammatory process that results in the development of non-healing chronic wounds. Bacterial infections, repeated episodes of ischemia injury and development of cellular and systemic reactions can ultimately result in hyper inflammation and pathological scar tissue formation (**Figure 1**). The ability to modulate the intensity and duration of the inflammatory response enabling the transition from an exudating wound to one that is the process of granulation holds promise to address these clinical problems and facilitate successful wound repair and regeneration.

**Figure 1:**
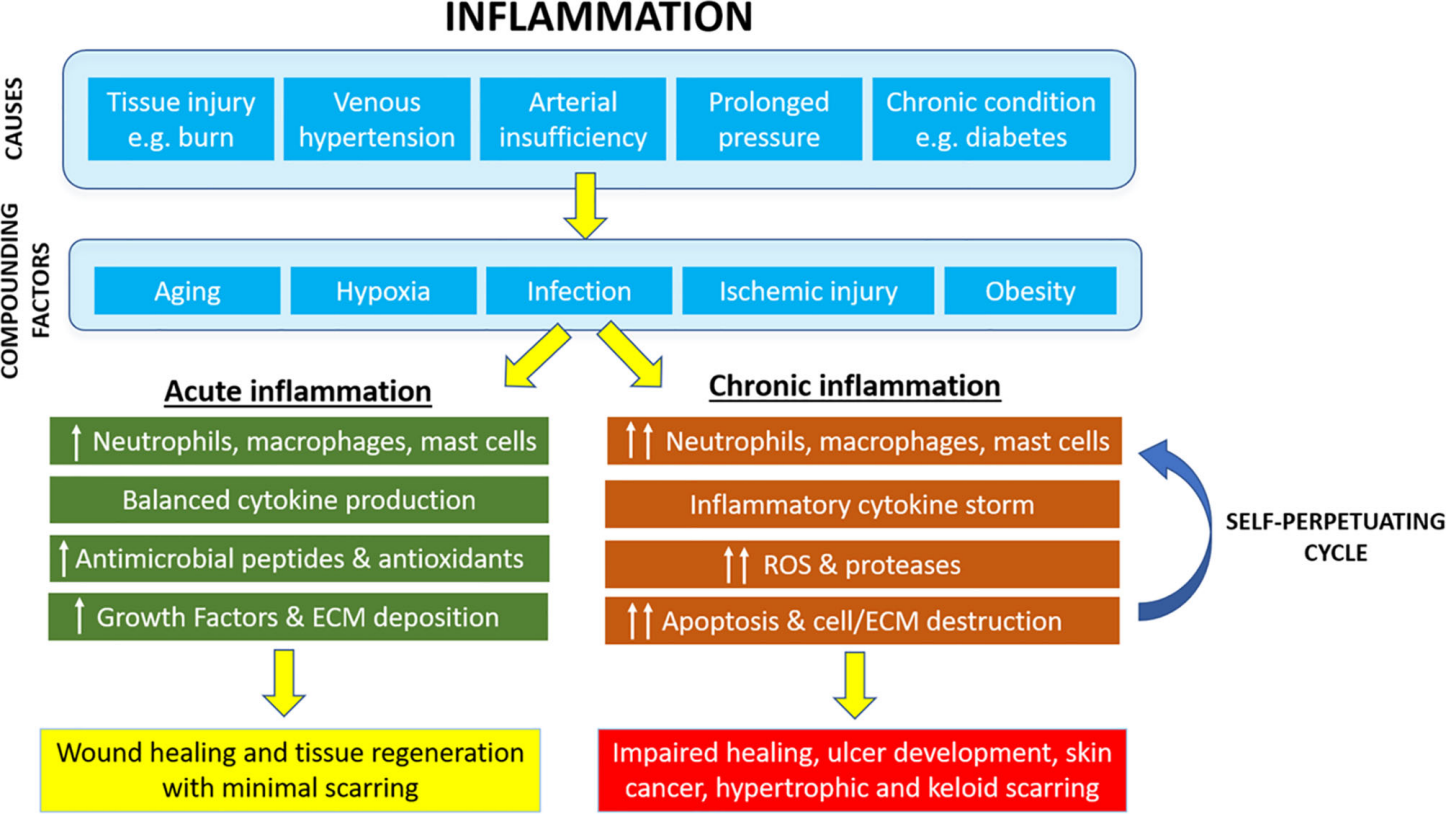
Cutaneous inflammation and its role in pathophysiology of chronic wounds and tissue regeneration.

Over the past few decades, scientific advances have greatly contributed to our current understanding of the complex molecular and cellular pathways that govern the natural progression of wound inflammation. The use of different *in-vitro* systems and experimental animal models has significantly improved our understanding of inflammatory pathways and the role of immune cells including T cells in the healing and regeneration of cutaneous wounds. These recent advances, as well as many novel and exciting approaches aimed at regulating inflammation, have made this a pertinent time for a Special Issue aimed at overviewing this important process. The goal of this Special Issue was therefore to provide a summary of the inflammatory regulation of healing and regeneration in cutaneous wounds and to provide further basic and translational research in this area to ultimately improve the treatment options for patients suffering impaired healing.

In this Research Topic, we welcomed four original articles and three reviews which discussed the role of inflammation in healing and regeneration of cutaneous wounds.

Oxidative stress has previously been associated with altered inflammation in chronic inflammatory disease leading to augmented restoration of skin homeostasis. An original article by Lee et al., described the an-inflammatory effect of liposomal astaxanthin antioxidant in reducing inflammatory cytokine release and oxidative stress in a murine model of atopic dermatitis demonstrating that liposomal formulation of astaxanthin enhances the therapeutic efficacy in modulating inflammation by improving transdermal delivery and highlighting the importance of balancing cytokine levels to restore skin homeostasis. This work was nicely complemented by a review on the role of Interlukin-22 in wound healing and tissue regeneration where Arshad et al., describe the double edged sword of IL-22 imbalance that governs both intricate innate immune responses that mediate inflammation, mucous production, and protection against pathogens during healing and tissue regeneration yet play a pivotal role in development of carcinogenesis. This highlighted the therapeutic perspective in modulating cytokine network to achieve wound healing, tissue regeneration and tumour prevention and treatment as well as importance in understanding the possible sources of IL-22 and signalling pathways involved in IL-22 regulation of inflammation during wound healing. The importance of modulating inflammatory cell levels, as mediators of inflammatory responses during wound healing and tissue regeneration, was also highlighted in a review by Wang et al., who described the role of inflammation in development of keloid and hypertrophic scars. Understanding the initiation, progression and resolution of inflammation holds promise to provide further insights into the mechanisms of scar formation as well as development of novel anti-scarring therapies. Article by Ud-Din et al., elegantly described the role of mast cells in skin scarring highlighting the need for further *in-vivo* human studies to define the exact mechanism by which mast cells influence scar formation and determine whether mast cells are a feasible target to reduce inflammation mediated scar formation and fibrosis.

In order to improve cutaneous healing and tissue regeneration, while limiting formation of hypertrophic scars an improved understanding of the immune responses induced by severe tissue injury is also required. A longitudinal clinical study by Mulder et al., characterized the systemic inflammatory response following severe burn wound injury by analysing the peripheral blood changes in subsets of innate and adaptive immune cells and inflammatory mediators over time to show that severe burn injury leads to persistent innate inflammatory response, including a release of immature neutrophils and a significant shift in effector and regulatory T cell composition towards a pro-inflammatory phenotype therefore contributing to systemic inflammation and increasing the risk of secondary complications including wound infection. Indeed, the clinical outcome of infected and non-infected wounds are widely different and the article by Hartman et al., used a combined peptidomic and bioinformatic approach to identify and characterise antimicrobial peptides and potential novel biomarkers in wound fluid of a small subset of patients with early wound infection diagnosis. This study illustrated that despite the small patient sample size, authors were able to identify large differences in peptide patterns demonstrating a power of combined peptidomics and bioinformatics approach for future research and clinical use. Lastly, research article by Niemiec et al., demonstrated the use of silk fibroin polymer in a nanosilk structure to effectively deliver anti-inflammatory microRNA-146a to diabetic murine wounds altering pro-inflammatory gene signalling and improve healing outcomes. Interestingly, the authors also demonstrated that treatment of human diabetic skin with 7% nanosilk solution strengthened the biomechanical properties promoting the pro-fibrotic processes and tissue regeneration.

In summary, this Research Topic provides a snapshot of the state of the field and provides new insights into the mechanisms and significance of inflammation in wound healing and tissue regeneration. Collectively the work highlights the imperative to further improve our understanding and delineate the precise mechanisms by which inflammation is generated, mediated, and regulated to open up opportunities to develop novel regenerative therapies to manipulate inflammatory responses and improve wound healing and clinical outcomes. By disseminating and sharing our research we ensure that the future advances in the filed can be made. These articles provide new knowledge that may lead to novel innovations to management of clinical inflammation and improve outcomes for people suffering from chronic wounds.

## Author Contributions

All authors listed have made a substantial, direct, and intellectual contribution to the work and approved it for publication.

## Funding

AJC is supported by the NHMRC Senior Research Fellowship (GNT #1102617). ZK is supported by the Channel 7 Children’s Research Foundation Fellowship and DEBRA Australia Research Grant.

## Conflict of Interest

AB is the founder and Scientific Director of Science of Skin.

The remaining authors declare that the research was conducted in the absence of any commercial or financial relationships that could be construed as a potential conflict of interest.

## Publisher’s Note

All claims expressed in this article are solely those of the authors and do not necessarily represent those of their affiliated organizations, or those of the publisher, the editors and the reviewers. Any product that may be evaluated in this article, or claim that may be made by its manufacturer, is not guaranteed or endorsed by the publisher.

